# The impact of educational interventional session on healthcare providers knowledge about pharmacovigilance at a tertiary Jordanian teaching hospital

**DOI:** 10.1186/s40545-023-00561-0

**Published:** 2023-04-13

**Authors:** Faris El-Dahiyat, Khawla Abu Hammour, Rana Abu Farha, Qusai Manaseer, Ala’a Momani, Aya Allan

**Affiliations:** 1 Clinical Pharmacy Program, College of Pharmacy, Al Ain University, Al Ain, United Arab Emirates; 2 AAU Health and Biomedical Research Center, Al Ain University, Abu Dhabi, United Arab Emirates; 3 Department of Biopharmaceutics and Clinical Pharmacy, School of Pharmacy, The University of Jordan, Amman, Jordan; 4 Clinical Pharmacy & Therapeutics Department, Faculty of Pharmacy, Applied Science Private University, Amman, Jordan; 5 Orthopedic Department, Jourdan University Teaching Hospital, Amman, Jordan; 6 Pharmacy Department, Jordan University Teaching Hospital, Amman, Jordan

**Keywords:** Pharmacovigilance, Jordan, Education

## Abstract

**Objective:**

A limited number of educational interventions among health care providers and students have been made in Jordan concerning the pharmacovigilance. Therefore, the main aim of this study was to evaluate how an educational workshop affected the understanding of and attitudes toward pharmacovigilance among healthcare students and professionals in a Jordanian institution.

**Methods:**

A questionnaire was used before and after an educational event to evaluate the pre- and post-knowledge and perception of pharmacovigilance and reporting of adverse drug reactions (ADRs) among a variety of students and healthcare professionals at Jordan University Hospital.

**Results:**

The educational workshop was attended by 85 of the 120 invited healthcare professionals and students (a response rate of 70.8%). The majority of respondents were capable of defining ADRs (*n* = 78, 91.8%) and pharmacovigilance accurately (*n* = 74, 87.1%) in terms of their prior understanding of the topic. Around 54.1% of the participants (*n* = 46) knew the definition of type A ADRs while 48.2% of them (*n* = 41) knew the definition of type B ADRs. Additionally, around 72% of the participants' believed that only serious and unexpected ADRs should be reported (*n* = 61, 71.8%), also, 43.5% of them (*n* = 37) believed that ADRs should not be reported until the specific medication that caused it is known. The majority of them (*n* = 73, 85.9%) agreed that reporting of ADRs was their responsibility. The interventional educational session has significantly and positively impacted participants' perceptions (*p* value ≤ 0.05). The most reason for not reporting ADRs as stated by the study participants was the lack of information provided by patients (*n* = 52, 61.2%) and the lack of enough time to report (*n* = 10, 11.8%).

**Conclusion:**

Participants’ perspectives have been greatly and favorably impacted by the interventional educational session. Thus, ongoing efforts and suitable training programs are required to assess the effect of bettering knowledge and perception on the practice of ADRs reporting.

## Introduction

By definition, pharmacovigilance is “the science and practices associated with the detection, assessment, understanding, and prevention of adverse drug reactions (ADRs) or any other drug-related problems,” as stated by the World Health Organization (WHO) [1]. According to reports, ADRs are almost the fifth-leading cause of death in the United States of America [2].

One of the major challenges worldwide is the underreporting of ADR [3]. Additionally, researchers indicated that the knowledge of health care providers on ADR reporting and pharmacovigilance was poor [4] that justify the results of previous research that indicated a low reporting rate of ADRs [5]. As a result, major ADRs may not be detected as soon as they should be.

In Jordan, the Department of Food and Drug Administration (JFDA) serves as Jordan's national drug regulatory body. JFDA was given the responsibility of coordinating with the WHO Program on behalf of about 130 other countries as a national PV center. To protect the population’s health, this program was started in Jordan in 2001. By compiling information from local PV centers in Jordan, it provides effective and secure medication.

One of these local PV centers is Jordan University Hospital. The efficiency of a PV program can be affected globally by the active engagement of healthcare professionals as well as their knowledge, attitude, and practice. Programs for education and training can raise their knowledge of PV and ADR reporting rates [3–6]. Also, the proficiency of healthcare professionals in the sector has had a substantial impact on the practice of pharmacovigilance. If properly instructed, there might be a strong incentive to improve reporting, which could improve the safety profiles of drugs. The interventional educational workshops may improve comprehension of a variety of health concerns, according to previous researches [5–8].

The results of this study can be used to determine the educational gap between healthcare professionals and students as well as the effects of educational initiatives that can support the promotion of safe practices and the PV environment among existing and future healthcare practitioners.

With this background, the current study aimed to assess how the educational workshop affected respondents’ knowledge of and attitudes about pharmacovigilance in a Jordanian tertiary teaching hospital and to assess the main barriers of reporting ADRs.

## Methods

### Settings and study subjects

This pre-post interventional study was conducted at the Jordan University Hospital (JUH), which is situated in Amman, Jordan. JUH is regarded as one of the first teaching hospitals at the level of the Arab World and the Middle East. It contains more than 25 specialty medical units and 620 beds. Sixty-four specialties and subspecialties in various medical fields are also included. The investigation was carried out by the pharmacy department, which was also managing a safety program as a component of the hospital's ongoing medical education. The program's objective is to inform pharmacists, nurses, and pharmacy students on various services relating to drug safety that were provided between July and October 2022. Three educational workshops were held to inform nurses, pharmacists, and pharmacy students about pharmacovigilance and the ADR reporting procedure throughout the study period. An invitation was sent to staff nurses, pharmacists and pharmacy students with a target of including 120 healthcare professionals and students, each session planned to serve 40 healthcare professionals. For the sample size calculation, a total of 107 are needed based on the following calculations. Researchers assumed that the knowledge and perception of participants on pharmacovigilance and reporting ADR to be 20%. Therefore, to get maximum possible size as follows:n = (Zα/2 + Zβ)2 ∗ (p1(1−p1) + p2(1−p2))/(p1-p2)2, where Zα/2 is the appropriate value from the normal distribution for the desired confidence interval. Zβ is the critical value of the normal distribution for the power β. p_1_ is the expected pre-intervention sample proportions. p_1_ is the expected post-intervention sample proportions. Using Zα/2 = 1.96 (95% confidence level), Zβ = 1.645 (95% power), p1 = 62.5% and p2 = 82.25%, with expected knowledge of 20% a minimum sample size of 107 healthcare providers was considered sufficient to obtain a significant difference between pre-intervention and post-intervention awareness about pharmacovigilance. A target sample size of 120 healthcare providers was approached to account for any drop-out after conducting the workshop session. healthcare professionals and students who fulfilled the inclusion criteria were approached and invited to participate in the study. Inclusion criteria were as follows: (1) experience of at least 2 years for health care providers and fifth- or sixth-year students who finished their hospital training module; (2) willing to participate in the study.

### Study questionnaire and scoring

With specific alterations made to fulfill the goal of this study, the study questionnaire was created and retrieved from other research studies that assessed healthcare practitioners’ knowledge, attitude, and practice toward pharmacovigilance [3, 5, 6]. Two academics with extensive backgrounds in this field of study conducted a peer assessment of the questionnaire. The questionnaire’s thoroughness and content clarity were evaluated (content validity). To assess the reliability of the questionnaire, a set of pharmacists were given the questionnaire, and the process was repeated later. The replies at the two time points were then compared, the percentages of agreement were calculated, and values at the two time points were compared. The questionnaire was divided into four sections, with either closed-ended or open-ended questions in each component. The sections covered the following topics: (1) features of healthcare providers' demographics; (2) understanding of pharmacovigilance, adverse drug reactions (ADRs), and the method for reporting them; (3) assessment of the significance of ADR reporting and who is in charge of doing so; and (4) their practice of reporting ADRs.

Eight questions were used to gauge respondents' understanding of the pharmacovigilance and ADR reporting procedures. Each answer was judged to be either right or wrong. Each accurate response earned one point, whereas each incorrect response resulted in a score of zero. Each respondent received an overall knowledge score out of eight.

### Study Procedures

Selected medical professionals and students totaling 120 were split into three groups of 40 each. Hospital employee affairs and the pharmacy school chose the students and healthcare professionals. As a result, three instructional workshop sessions covering the three groups were planned. Two trained members of staff—a pharmacist and a PharmD—who had received training in how to deliver the study questionnaires oversaw the study session. Healthcare professionals and students were asked to complete the questionnaire before starting the session of the workshop. They had 10 min to do so before returning it to the staff. This was the pre-intervention baseline data. Healthcare professionals were given post-intervention questionnaires as soon as the intervention session ended, and they had an additional 10 min to fill them out and return them.

### Educational workshop

The educational pharmacovigilance training lasted for one hour. The head of the pharmacy department at JUH prepared and delivered a power point presentation during the workshop. The main goal of this training was to increase healthcare professionals' and students' awareness of pharmacovigilance and the procedure of reporting the ADR. The educational program included an overview of pharmacovigilance, an explanation of how to recognize ADRs, information on the different categories of ADRs, information on the yellow form and the electronic form used to report ADRs, and details on the reporting procedure. After this informative discussion, there was an opportunity for participants to ask questions.

### Ethical considerations

The study was conducted after receiving ethical approval from the institutional review board at JUH (Reference number: 10/4022/8379). The World Medical Association Declaration of Helsinki guidelines for ethical conduct were followed during the study’s conduction [9]. Each subject gave verbal informed consent before to the study's start. Participants were informed of the study's voluntary nature and given the option to leave before completing the post-workshop questionnaire. Participants were also informed that their responses would be kept confidential and used only as part of a cohort for analysis.

### Statistical analysis

The data were examined using SPSS version 22 (Statistical Package for Social Science) (SPSS Inc., Chicago, IL, USA). The descriptive analysis employed percentage for qualitative variables and mean and standard deviation (SD) for continuous variables. McNemar’s test was used to evaluate differences in categorical variables between pre- and post-workshop data. A paired t-test was carried out to evaluate changes between pre- and post-test knowledge score (continuous data). A P-value of less than 0.05 was considered statistically significant for all two-tailed tests and statistical analyses.

## Results

Only 85 of the 120 healthcare professionals and students who were invited to the workshop’s various sessions actually attended (response rate: 70.8%). All of the participants completed the questionnaire before and after the intervention. Thirty-nine of them (45.39%) were pharmacists, 29 (34.11%) were nurses, and 17 (20%) were pharmacy students. Table 1 details the demographics of the respondents, showing that the majority (*n* = 130, 86.7%) were under the age of 40 and that female made up 40% (*n* = 60) of them.

**Table 1:** The sociodemographic of the study sample (*n* = 85)

Variable	n (%)
*Age*	
18–25 years	25 (29.8)
26–36 years	33 (39.3)
36–45 years	21 (25.0)
> 45 years	6 (7.1)
Gender	
Males	22 (26.2)
Female	63 (73.8)
Healthcare provider category	
Pharmacists	39 (45.9)
Pharmacy students	17 (20.0)
Nurses	29 (34.1)
Attending previous pharmacovigilance workshop	
Yes	55 (65.5)
No	29 (34.5)

Table 2 also includes responses to numerous questions being asked to healthcare professionals before and after the intervention. The majority of respondents were capable of defining ADRs (*n* = 78, 91.8%) and pharmacovigilance accurately (*n* = 74, 87.1%) in terms of their prior understanding of the topic. Around 54.1% of the participants (*n* = 46) knew the definition of type A ADRs while 48.2% of them (*n* = 41) knew the definition of type B ADRs. Additionally, around 72% of the participants' believed that only serious and unexpected ADRs should be reported (*n* = 61, 71.8%), also, 43.5% of them (*n* = 37) believed that ADRs should not be reported until the specific medication that caused it is known. Table 2 shows that participants’ understanding of the pharmacovigilance and ADRs reporting process was significantly improved following the education workshop except for four questions/statements, where there were no significant variations between the pre- and post-workshop response (*P*-value > 0.05).

**Table 2:** The impact of educational workshop on healthcare providers’ understanding of pharmacovigilance and the ADRs reporting system (*n* = 85)

Questions	The correct answer	Frequency and percentage of participants answered correctly		
		Pre-workshop	Post-workshop	*P*-value^a^
Definition of ADRs	ADR is a response to a drug which is noxious and unintended, and which occurs at doses normally used in man for the prophylaxis, diagnosis, or therapy of disease, or for the modifications of physiological function	78 (91.8)	80 (94.1)	0.530
Definition of pharmacovigilance	Pharmacovigilance is the science and activities relating to the detection, assessment, understanding and prevention of adverse effects or any other drug-related problem	74 (87.1)	85 (100.0)	0.001*
Definition of ADR type A	A reaction that is predictable from the known pharmacology of a drug and are associated with high morbidity and low mortality	46 (54.1)	74 (87.1)	< 0.001*
Definition of ADR type B	A reaction that is idiosyncratic, and cannot be predicted from the known pharmacology of a drug and are associated with low morbidity and high mortality	41 (48.2)	65 (76.5)	< 0.001*
ADR should be reported only when serious and unexpected	No	61 (71.8)	67 (78.8)	0.320
Always report adverse effects related to herbal medications	Yes	70 (82.4)	75 (88.2)	0.227
ADR should not be reported until the specific medication that caused it is known	No	37 (43.5)	48 (56.5)	0.101
Location of the national pharmacovigilance center in Jordan	Yes	48 (56.5)	76 (89.4)	< 0.001*
Overall knowledge score (mean ± SD)		5.6 ± 1.9	6.6 ± 1.1	< 0.001^b^*

*significant at 0.05 significance level

^a^Using McNemar test

^b^Using paired *t*-test

Table 3 displays respondents’ perspectives on the significance of reporting adverse ADRs, whether healthcare professionals and students are accountable for reporting these ADRs, and how thoroughly pharmacovigilance science should be taught. The majority of them (*n* = 73, 85.9%) agreed that reporting of ADRs was their responsibility. The interventional educational session has significantly and positively impacted participants' perceptions, as shown in Table 3, which includes statements like “I believe that I am sufficiently knowledgeable to report ADRs in my future practice” and “I believe that my profession is one of the most important professions to report ADRs” (*p* value ≤ 0.05).

**Table 3:** The impact of educational workshop on healthcare providers’ perceptions of toward ADR reporting (*n* = 85)

Statement	Pre-workshop	Post-workshop	P-value^a^
I think reporting of ADR is necessary	82 (96.5)	84 (98.8)	0.320*
I believe that healthcare professionals should be thoroughly instructed in pharmacovigilance	77 (90.6)	81 (95.3)	0.251
I have read at least one article on prevention of adverse drug reactions	47 (55.3)	50 (58.8)	0.664
I think it is important to establish an ADR monitoring center in every hospital	75 (88.2)	83 (97.6)	0.020*
The subject of pharmacovigilance ought to be taught as a key subject in the curriculum	70 (82.4)	80 (94.1)	0.018*
I believe my degree program adequately covers the subject of ADRs	38 (44.7)	45 (52.9)	0.288
I really believe that it is my duty as a professional to report ADR	73 (85.9)	80 (94.1)	< 0.001*
ADRs that have previously been reported would not significantly improve the reporting mechanism	46 (54.1)	53 (62.4)	0.288
I believe that I am sufficiently knowledgeable to report ADRs in my future practice	46 (54.1)	72 (84.7)	< 0.001*
I believe that my profession is one of the most important professions to report ADRs	75 (88.2)	83 (97.6)	0.020*
I believe ADR reporting should be made compulsory for all health care Professionals	82 (96.5)	82 (96.5)	< 0.001*
Concerned officials are not actively trying to enhance Jordan's ADR reporting system	45 (52.9)	46 (54.1)	0.877

*significant at 0.05 significance level

^a^Using McNemar test

Table 4 reveals that ADRs have only ever been reported by less than one-third of the study participants (24, 28.2%). The most reason for not reporting ADRs as stated by the study participants was the lack of information provided by patients (*n* = 52, 61.2%) and the lack of enough time to report (*n* = 10, 11.8%). Other reasons for not disclosing ADRs are reported in Table 4.

**Table 4:** The practice of healthcare professionals with regard to ADR reporting pre-workshop (*n* = 85)

Questions	n (%)
Have you seen an adverse drug reporting system?	
Yes	40 (47.1)
No	45 (52.9)
Have you ever visited any ADR monitoring center?	
Yes	12 (14.1)
No	73 (85.9)
Have you ever reported any ADR?	
Yes	24 (28.2)
No	61 (71.8)
Reasons for not reporting ADRs	
Lack of information provided by patients	52 (61.2)
I don't have enough time	10 (11.8)
I am not sure how and where to report	9 (10.6)
I don't think it's important	2 (2.4)
The appropriate authorities do not actively promote it	4 (4.7)
I worry about having legal issues	7 (8.2)
Missing data	1 (1.2)

## Discussion

Recently, pharmacovigilance has gained significance as a crucial component of efficient medication control systems, clinical practice, and public health initiatives [10]. While spontaneous reporting is still a crucial tool in discovering and reporting ADRs to minimize injury, healthcare professionals play a significant role in this process [11]. For that reason, it was essential to carry out thorough research to investigate and assess the roles and contributions of healthcare providers and students in the pharmacovigilance operation of the system.

Numerous studies have been conducted in Jordan assessing the perceptions and understanding of healthcare professionals in regard to pharmacovigilance, but few training or interventions have been made [3–6]. Prior to the training, the participating healthcare professionals and students in this study had an acceptable knowledge score (score = 5.6/8). In contrast to findings from other studies showed that most healthcare practitioners were not familiar with the term pharmacovigilance [3, 5, 12, 13]. In terms of understanding what pharmacovigilance is, our respondents were the most knowledgeable (87.1%). In terms of understanding what ADR type B is, our respondents were the least aware where around half of the respondents had the right response. These findings coincided with those of a study carried out in Kuwait. The majority of pharmacists exhibited good awareness of the principle of pharmacovigilance and ADRs in terms of their concepts and objectives, according to researchers who evaluated pharmacists' knowledge and perception of pharmacovigilance and ADR reporting [14]. However, findings from a research conducted in Arabian country revealed that few medical professionals were aware of the existence of a national pharmacovigilance center [18].

It is important to underline the significance of ADR reporting. According to earlier studies, developing methods that aim to optimize both knowledge and practices with relation to pharmacovigilance could boost the reporting of ADRs [16]. Since knowledge and awareness of the complete pharmacovigilance system have an impact on practice, an earlier study unequivocally showed that Jordanian healthcare providers had inadequate ADR reporting practice [3]. The fact that the existing pharmacovigilance programs in the Middle Eastern region are still in their early stages contributes validity to these findings [17].

It is also worth mentioning that due to a lack of funding to promote enrollment in pharmacovigilance professional development courses, low- and middle-income nations have a very low proportion of health workers with drug safety capabilities [18]. As a result, there are now fewer professionals in underdeveloped countries who can evaluate the safety of drugs and enhance risk management [18, 19].

The results of the current investigation demonstrated an immediate, significant improvement in healthcare providers' knowledge scores following the educational workshop. It was not unexpected because the respondents had already received the relevant information about pharmacovigilance. Similar results were obtained by earlier research [6, 20, 21], with the only difference being the time period. Additionally, a study done in Nigeria revealed that pharmacovigilance training for medical practitioners had a significant impact on both knowledge and practice ratings [22]. Another Indian study also revealed that doctors who participated in pharmacovigilance medical education were more knowledgeable about the ADR reporting system than their non-participating counterparts [23].

Moreover, healthcare professionals demonstrated a favorable attitude toward the obligation to report ADRs and the significance of this reporting pre-workshop. This educational workshop produced yet another notable improvement in participants’ perception score across the board. The degree to which people express favorable or negative feelings about particular behaviors or practices is known as perception [24]. According to the theory of planned behavior, perception is one of the major determinants of people's conduct, along with intention, subjective norm, and perceived behavioral control [25].

The intentions of healthcare professionals to engage in various actions are also found to be well-predicted by perception or attitude [26–28]. The necessity of concentrating on healthcare providers' attitudes to increase their intention to report ADRs can be justified by the well-established knowledge of the impact of perception on intended behaviors among healthcare providers.

In this study, the effect of the educational intervention on the practice of ADR reporting was not investigated, despite the initial improvement in healthcare providers' knowledge of and attitudes toward the pharmacovigilance system following the educational intervention. An earlier study from India found that pharmacovigilance education produced appropriate knowledge and favorable views about pharmacovigilance and ADR reporting, but that healthcare providers continued to ignore the practice of ADR reporting [23].

Additionally, a German study found that the impact of educational interventions on pharmacovigilance had only a transient impact on healthcare practitioners' ADR practices [30]. However, a prior analysis from Nigeria revealed that the conclusion of training workshops with a lecture-based format led to a little rise in the quantity of ADRs that were reported [22]. According to previous researches, knowledge and attitudes are considered modifiable elements that appear to be strongly connected with reporting practice [31, 32].

The use of a self-rated evaluation method, where healthcare practitioners might have inflated their perception level, is one of the study's major flaws. Additionally, the impact of the educational workshop was examined just after the workshop, which might not accurately reflect the effect over the long term. Therefore, additional research may be required to assess the influence of educational workshops on the long-term impacts following the implementation of the intervention. This study included healthcare professionals who worked at a single institution. As a result, the findings of this study might not apply to all healthcare centers in Jordan.

## Strengths and limitations

This is the first study to evaluate the effectiveness of educational intervention, including both students and healthcare professionals. However, before drawing conclusions from the results, a few limitations should be kept in mind. First, despite the initial improvement in healthcare professionals' understanding and perception of the pharmacovigilance system following the educational intervention, this educational intervention's impact on the practice of ADR reporting was not investigated. The fact that the effects of the educational workshop were studied immediately after the workshop may not accurately reflect their effect in the long term, which is another one of the study's major limitations. This study included students and healthcare professionals from a single institution. So, it is possible that the findings of this study will not apply to other Jordanian institutions.

## Conclusion

The main findings of this study showed that health care providers and students' perspectives have been positively impacted by the interventional educational session. Thus, ongoing efforts and suitable training programs are required to assess the effect of better knowledge and perception on the practice of ADRs reporting.

## Data Availability

The data that support the findings of this study are available from the corresponding author upon reasonable request.
